# Assessment of Fetal Autonomic Nervous System Activity by Fetal Magnetocardiography: Comparison of Normal Pregnancy and Intrauterine Growth Restriction

**DOI:** 10.1155/2011/218162

**Published:** 2011-04-11

**Authors:** Akimune Fukushima, Kenji Nakai, Tomonobu Kanasugi, Miyuki Terata, Toru Sugiyama

**Affiliations:** ^1^Department of Obstetrics and Gynecology, School of Medicine, Iwate Medical University, Morioka 020-8505, Japan; ^2^Department of Dentistry and Internal Medicine, Iwate Medical University, Morioka 020-8505, Japan

## Abstract

*Objective*. To clarify the developmental activity of the autonomic nervous system (ANS) of the normal fetus and intrauterine growth restriction (IUGR) cases using fetal magnetocardiography (FMCG). *Subjects and Methods*. Normal pregnancy (*n* = 35) and IUGR (*n* = 12) cases at 28–39 and 32–37 weeks of gestation, respectively, were included in this study. The R-R interval variability was used to calculate the coefficient of variance (CV_RR_) and low frequency/high frequency (LF/HF) ratio. *Results*. The value of CV_RR_ in the normal pregnancy group displayed a slight increasing trend with gestational age. However, no such trend was observed in the IUGR group. In contrast, the LF/HF ratio in both the normal pregnancy group and the IUGR group clearly increased over the gestational period; the normal group showing statistical significance. *Conclusion*. The development of fetal ANS activity in IUGR cases might differ from that observed in the normal pregnancy group, and this may facilitate early detection of IUGR.

## 1. Introduction

Intrauterine growth restriction (IUGR) delays or terminates intrauterine fetal development; abnormal fetal condition is displayed as the pregnancy progresses. Numerous factors are involved in IUGR. Besides the frequently encountered high-risk cases of pregnancies and parturitions, neurological complications and fetal mortalities are also known to correlate closely with IUGR [1–6]; therefore, early detection and causative reference of IUGR are critical in assessing fetal well-being. Although deficient in certain aspects, routine fetal monitoring is currently performed using ultrasonography and cardiotocography. Magnetocardiography (MCG) is a safe and noninvasive method that employs a superconducting quantum interference device (SQUID) to facilitate a 3-dimensional analysis of the micromagnetic field (10^−12^ tesla) generated by phenomenal electric activities of the heart. Recent applications of fetal MCG (FMCG) have yielded details of acquired QT prolongation syndrome, supraventricular tachycardia, and various fetal arrhythmias [7–14].

Attention has recently been focused on spectral analysis of heart rate variability as a method for evaluating fetal well-being; for example, the efficacy of analyses of the coefficient of R-R interval variability (CV_RR_), low-frequency (LF) and high-frequency (HF) domains, and the LF/HF ratio in a supine resting posture have been suggested for the evaluation of autonomic nervous system (ANS) activities [15, 16]. The methods of ultrasonography and cardiotocography, which are incapable of measuring CV_RR_, LF/HF ratio, and various fetal heart rate variability analyses, can be improved with FMCG, thereby enabling these indices to be determined. We previously reported that sympathetic nervous activity increased with gestational age in the normal pregnancy [17]. In the present study, we evaluated the actual autonomic nervous system development of normal fetuses at 28–37 weeks of gestation. In the present study, differences of actual autonomic nervous system development between normal and IUGR cases were compared using FMCG.

## 2. Materials and Methods

### 2.1. Subjects

Subjects enrolled in the present study were women with normal pregnancies (*n* = 35) at 28–39 weeks of gestation and those with IUGR (*n* = 12) at 32–37 weeks of gestation that visited our hospital as either outpatients or inpatients between January 2004 and July 2005 (Table 1). In the normal pregnancy group, fetuses were born at term and without any abnormal neurological signs. In the IUGR group, ultrasonography was performed by several specialists for all cases who underwent FMCG. All of these cases presented with asymmetrical IUGR with weight 10% less than normal values; none had complications at birth.

Written informed consent was obtained from all subjects after being briefed about the clinical study, which was approved by the Ethics Committee of the School of Medicine, Iwate Medical University (H14-33, H17-2).

### 2.2. Equipment

**Figure 1:**
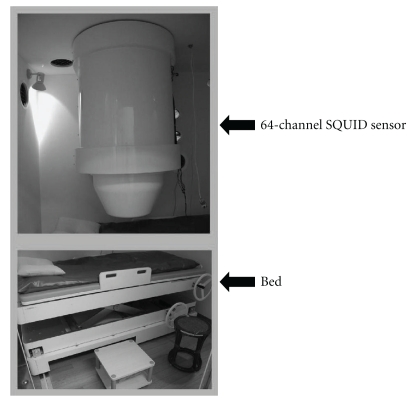
A monitoring device for magnetocardiography (MCG) incorporating a 64-channel SQUID apparatus in a magnetically shielded room.

**Figure 2:**
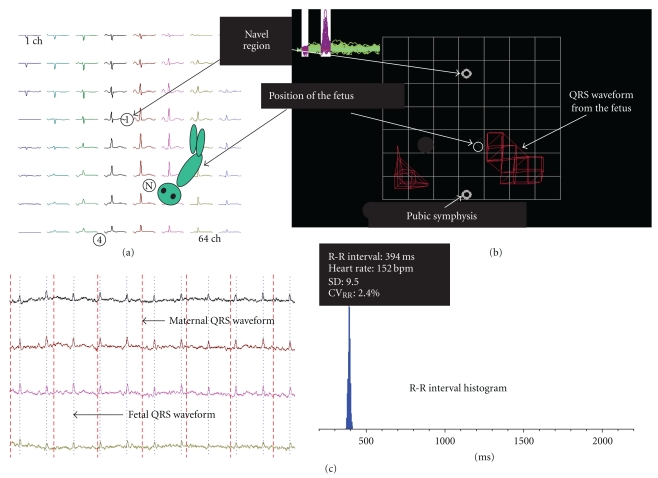
Method of fetal magnetocardiography (FMCG). The position of the fetus was referenced using standard coordinates at the navel region and pubic symphysis (a). Detected magnetic field signals, which comprised signals derived from both the mother and fetus, were subtracted from the maternally derived QRS waveform (b). In performing FMCG, a total of 300–500 heartbeats detected per determination were summated to yield the mean (c).

**Figure 3:**
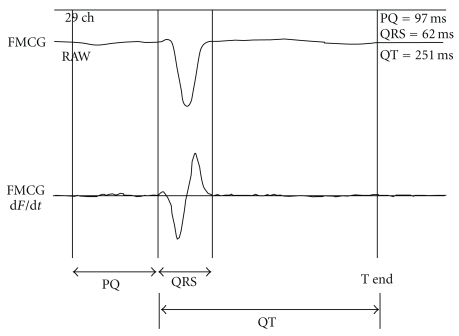
Determination of PQ, QRS, and QT intervals by differentiation. In performing FMCG, a total of 300–500 heartbeats detected per determination were summated to yield the mean. The initial and termination points of the PQ, QRS, and T wave were defined by the first derivate of d*F*/d*t*.

To obtain FMCG measurements, pregnant volunteers assumed a supine position on a bed and were scanned with a 64-channel MCG sensor (prototype made by the Joint Research Project for Regional Intensive in Iwate Prefecture; SQUID sensor, Hitachi High-Technology Co. Ltd., Tokyo, Japan) installed in a magnetically shielded room (Figure 1). The position of the fetus was determined with ultrasonography, using the navel and pubic symphysis as reference coordinates (Figures 2(a) and 2(b)). The magnetic field in the z direction (Bz) adjacent to the body surface was monitored continuously for 5 minutes. A total of two or three measurements were obtained in this manner. Because the detected Bz is a summation of signals from fetal and maternal sources, the maternal QRS waveform was subtracted from the detected Bz to derive the actual Bz value. Fetal heart rhythm (approximately 700 beats) was measured for 5 minutes, and the average heart rate was used to derive the FMCG in each case (Figure 2(c)). The initial and termination values of PQ, QRS, and QT were defined by the maximum points of the first derivate of d*F*/d*t* (Figure 3). CV_RR_ was calculated as follows:

(1) $$\begin{array}{c} \text{Standard}\;\text{deviation}\;\begin{array}{c} \left(\text{SD}\right)=\sqrt{\frac{1}{n}\overset{n}{\underset{i=1}{\sum }}{\left(Di-\bar{M}\right)}^{2}} \end{array},\\ {\text{CV}}_{\text{RR}}=\frac{\text{SD}}{\bar{M}}\times 100\left(\%\right), \end{array}$$

where *n* and *Di* represent the number of R-R intervals and the mean of approximately 700 R-R intervals, respectively, and $\bar{M}$ represents the total mean of the respective *Di*.

The power spectrum in the frequency domain was derived from frequency-field components using the maximum entropy method of fetal heart rate variability. Based on frequency analysis, the ranges of the LF and HF domains were defined as 0.01–0.15 and 0.15–0.4 Hz, respectively. The respective power values were derived to yield the LF/HF ratio. Note that the LF/HF ratio is taken as a sympathetic activity of ANS [18, 19].

### 2.3. Statistical Analysis

Data were analyzed using StatView for Windows Ver. 5.0 (SAS Institute Inc., Cary, NC, USA). The relationships among CV_RR_, LF/HF, and gestational age in each group were analyzed by linear regression, while intergroup changes in CV_RR_ and LF/HF over the gestational period in each group were verified by one-way ANOVA. Changes in CV_RR_ and LF/HF over the gestational period between normal pregnancy and the IUGR groups were analyzed by two-way ANOVA.

## 3. Results

The normal pregnancy group was divided into three groups for classifying one-way ANOVA analysis of CV_RR_ and LF/HF as follows: Group (A): 28–31 weeks of pregnancy (8th month of pregnancy); Group (B): 32–35 weeks of pregnancy (9th month of pregnancy); Group (C): 36–40 weeks of pregnancy (10th month of pregnancy).

**Figure 4:**
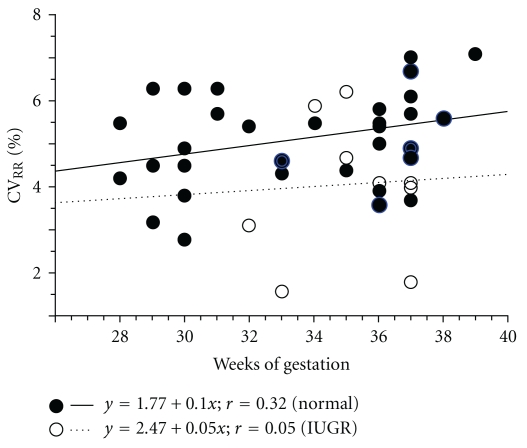
Correlation between the coefficient of variance (CV_RR_) and gestational age in normal pregnancy and in IUGR (normal pregnancy: ●—; IUGR: ∘…).

**Figure 5:**
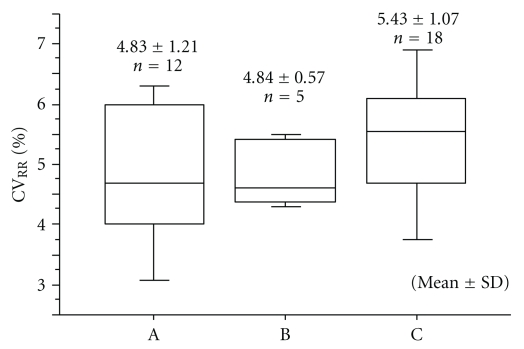
Intergroup changes in the coefficient of variance (CV_RR_) during normal pregnancy. Group (A): 28–31 weeks of pregnancy; Group (B): 32–35 weeks of pregnancy; Group (C): 36–40 weeks of pregnancy. One-way ANOVA: *P* = 0.28 [17].

The value of CV_RR_ in normal pregnancy showed a slight increased trend with gestational age (*y* = 1.77 + 0.10*x*; *r* = 0.32) (Figure 4). In contrast, the value of CV_RR_ in the IUGR group showed no such trend (Figure 4). Intergroup changes in CV_RR_ over the gestational age periods in normal pregnancy showed no statistical difference by one-way ANOVA [17] (Figure 5).

**Figure 6:**
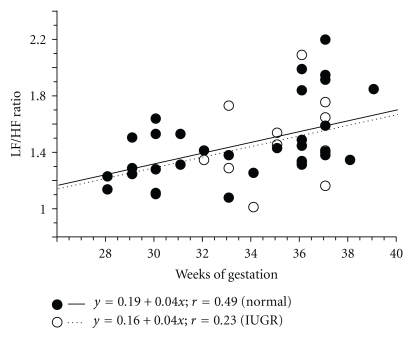
Correlation between the low frequency/high frequency (LF/HF) ratio and gestational age in normal pregnancy and IUGR. (Normal pregnancy, ●—; IUGR, ∘…).

**Figure 7:**
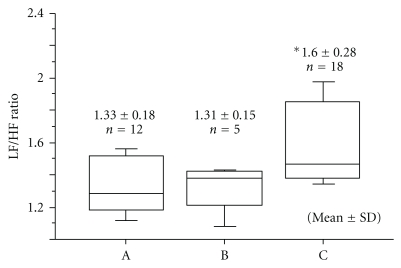
Intergroup changes in the low frequency/high frequency (LF/HF) ratio during normal pregnancy. Group (A): 28–31 weeks of pregnancy; Group (B): 32–35 weeks of pregnancy; Group (C): 36–40 weeks of pregnancy. *One-way ANOVA: *P* = 0.003 [17].

The value of LF/HF in both the normal pregnancy group and the IUGR group showed an increase with gestational age (*y* = 0.19 + 0.04*x*, *r* = 0.49; *y* = 0.16 + 0.04*x*, *r* = 0.23, resp.) (Figure 6). Intergroup changes in LF/HF in the normal group increased significantly according to the gestation period (one-way ANOVA: *P* = 0.003) [17] (Figure 7). There was no statistical difference in intergroup changes for the LF/HF ratio between normal pregnancy and IUGR groups.

## 4. Discussion

Recent advances in medical electronics have enabled various fetal parameters in the field of perinatal medicine to be obtained in greater detail and with higher precision. Current improved versions of ultrasonography devices can provide detailed evaluation regarding morphology and/or blood flow dynamics. To assess fetal ANS function, Shields and Schifrin evaluated fetal heart rate variability derived from fetal cardiotocography and respiratory movements [20]. More than 90% of fetal asphyxia cases were detected by abnormal heartbeat patterns using cardiotocography. Thus, cardiotocography is an indispensable tool that is routinely employed in clinical obstetrics; however, even when abnormal heartbeat patterns are detected, the combined use of ultrasonic Doppler and biophysical profile analysis does not improve the ability to identify undiagnosed cases of asphyxia. These conventional methods are yet to objectively and reliably evaluate functional development of the fetal autonomic nervous system; they are also unable to consistently achieve prenatal diagnosis of conditions that result from abnormal fetal autonomic nervous system development, such as cerebral palsy. As a result, unnecessary obstetric interventions are encountered, and the rate of cesarean section continues to increase without any decrease in the incidence of fetal central nervous system impairments, especially cerebral palsy. This problem indicates the need for a novel method for prenatal diagnosis [21, 22].

In the present study, we developed an FMCG by modifying our recently developed 64-channel MCG [23, 24]. The special features of MCG make it possible for the device to perform a 3-dimensional analysis of the magnetic field generated by the phenomenal electric activities of the heart in both fetuses and adults. FMCG is an extremely safe and noninvasive method for monitoring fetal cardiac activities [8]. To date, numerous studies have used FMCG to analyze fetal arrhythmias [11, 13, 14, 25–27]. Although analyses of heart rate variability have been attempted on fetal autonomic nervous system functions using FMCG, little has been established using this technique [28, 29].

In the present study, FMCG signals were generally too weak to enable a precise analysis of fetal ANS activity to be performed prior to 28 week of pregnancy in the normal pregnancy group and prior to 32 weeks of pregnancy in the IUGR group. To advance the development of this method, we undertook the following studies: (i) elucidation of the developmental stages of the fetal autonomic nervous system over a period of 39 weeks and (ii) comparison of FMCG findings between normal pregnancy and IUGR groups. In IUGR cases, both the difference in fetal size and other obstetric risks are higher, and the correlation dimension decreases when compared with the normal pregnancy group; that is, the heart rate coordination system is nonversatile and highly susceptible to stress [30, 31]. In brief, it is critical to differentiate on grounds of perinatal management whether a fetus has low body weight alone or whether it also has endogenous functional issues.

According to our previous study [17], the result of measurements attempted on CV_RR_, a value that reflects parasympathetic nervous system (PSN) activity [15, 16], indicated that CV_RR_ values exhibited a slight increasing trend with gestational age in the normal pregnancy group. In addition, we defined a value of the LF/HF ratio as the sympathetic nervous system (SNS) activity or balancing factor between the SNS and the PNS activities [29]. The LF/HF ratio showed a clear increase with gestational age in the normal pregnancy group [17]. It is understood that fetal ANS function develops as gestation progresses [29]. These results are consistent with ultrasound analyses of the fetal mouthing movement interval and eye-movement phase patterns, in which major changes are seen to occur at around gestational weeks 28–33 [31]. This evidence from earlier studies is consistent with the results of the present study. Our results revealed that the maturation process, especially in SNS activity or the balancing factor between SNS and PNS activities, changes dramatically from 28–39 weeks gestation. For the fetus, therefore, it is very important to avoid preterm delivery, not only in terms of body size and organic maturation but also for the functional development of ANS.

A previous study obtained by frequency analysis using FMCG indicated decreased complexity and increased periodicity of the LF and HF ranges in IUGR cases [27]. In the present study, the value of the LF/HF ratio in the IUGR group tended to increase with gestational age; however, the value of CV_RR_ did not show a clear trend. This finding might depend on growth restriction or heterogeneity in terms of etiology and severity in the IUGR cases [32]. Further investigation of IUGR cases, along with detailed analyses of patient background, is therefore required. The development pattern of ANS in the fetal period may become one of the important indices for prenatal management in the near future.

## Figures and Tables

**Table 1:** Descriptive characteristics of normal and intrauterine growth restricted (IUGR) fetuses.

Item	Normal fetuses (*n* = 35) (max–min)	IUGR fetuses (*n* = 12) (max–min)	*P* value
Maternal age (years)	31.7 ± 6.46 (44–20)	30.9 ± 5.79 (42–23)	NS
Gestational age (weeks)	33.9 ± 3.49 (39–28)	35.2 ± 1.80 (37–32)	NS

Values are represented as the mean ± SD. NS: not significant.
